# Insufficient Chilling Effects Vary among Boreal Tree Species and Chilling Duration

**DOI:** 10.3389/fpls.2017.01354

**Published:** 2017-08-15

**Authors:** Rongzhou Man, Pengxin Lu, Qing-Lai Dang

**Affiliations:** ^1^Ontario Ministry of Natural Resources and Forestry, Ontario Forest Research Institute, Sault Ste. Marie ON, Canada; ^2^Faculty of Natural Resources Management, Lakehead University, Thunder Bay ON, Canada

**Keywords:** chilling requirement, heat accumulation, dormancy release, climatic warming, spring bud phenology

## Abstract

Insufficient chilling resulting from rising winter temperatures associated with climate warming has been an area of particular interest in boreal and temperate regions where a period of cool temperatures in fall and winter is required to break plant dormancy. In this study, we examined the budburst and growth of trembling aspen (*Populus tremuloides* Michx.), balsam poplar (*Populus balsamifera* L.), white birch (*Betula papyrifera* Marsh.), black spruce (*Picea mariana* (Mill.) B.S.P.), white spruce (*Picea glauca* (Moench) Voss), jack pine (*Pinus banksiana* Lamb.), and lodgepole pine (*Pinus contorta* Dougl. ex. Loud.) seedlings subjected to typical northern Ontario, Canada, spring conditions in climate chambers after different exposures to natural chilling. Results indicate that chilling requirements (cumulative weighted chilling hours) differed substantially among the seven species, ranging from 300 to 500 h for spruce seedlings to more than 1100 h for trembling aspen and lodgepole pine. Only spruce seedlings had fulfilled their chilling requirements before December 31, whereas the other species continued chilling well into March and April. Species with lower chilling requirements needed more heat accumulation for budburst and vice versa. Insufficient chilling delayed budburst but only extremely restricted chilling hours (<400) resulted in abnormal budburst and growth, including reduced needle and shoot expansion, early budburst in lower crowns, and erratic budburst on lower stems and roots. Effects, however, depended on both the species’ chilling requirements and the chilling–heat relationship. Among the seven tree species examined, trembling aspen is most likely to be affected by reduced chilling accumulation possible under future climate scenarios, followed by balsam poplar, white birch, lodgepole pine, and jack pine. Black and white spruce are least likely to be affected by changes in chilling hours.

## Introduction

In temperate and boreal climates, winter dormancy is a critical adaptation of plants that prevents precocious spring development when conditions are not conducive to active growth (Heide, 2003; Vitasse et al., 2014). After growth stops and bud dormancy is induced by shorter photoperiods in the late summer and early fall, a period of cool temperatures (chilling) is required for plants to break dormancy (Saxe et al., 2001; Kalberer et al., 2006; Polgar and Primack, 2011). Without adequate chilling, budburst can be delayed (Cannell and Smith, 1983; Myking and Heide, 1995; Heide, 2003; Morin et al., 2009) and subsequent shoot growth compromised (Byrne and Bacon, 1992; Myking and Heide, 1995; Arora et al., 2003; Laube et al., 2014). While the focus of chilling research in woody plants has been largely on timing of budburst (Cannell and Smith, 1983; Morin et al., 2009; Polgar and Primack, 2011; Polgar et al., 2014), rarely have the effects of insufficient chilling on quality of budburst and subsequent growth been examined, especially in forest tree species for which foliage abundance and shoot growth have significant ecological and economic importance (Polgar and Primack, 2011).

After the chilling requirement is fulfilled (dormancy release), a certain accumulation of heat is needed to force budburst (Saxe et al., 2001; Heide, 2003; Polgar and Primack, 2011). To some extent, insufficient chilling can be compensated by additional heat, without compromising budburst (Cannell and Smith, 1983; Heide, 1993; Hannerz et al., 2003; Guak and Neilsen, 2013). The chilling–heat relationship, however, has been quantified for only a few tree species (Cannell and Smith, 1983; Heide, 1993; Hannerz et al., 2003; Harrington et al., 2010), and little is known about the range of chilling levels required to ensure budburst and growth are not negatively affected.

Several models have been developed to quantify chilling accumulation. The simplest model accounts only for the number of days or hours within the range of optimum chilling temperatures (Weinberger, 1950; Cannell and Smith, 1983). More complicated models take into consideration the differential effects of chilling in a broader range of temperatures that may contribute positively (Landsberg, 1974; Sarvas, 1974; Hänninen, 1990; Hannerz et al., 2003) or negatively (Richardson et al., 1974; Byrne and Bacon, 1992; Guak and Neilsen, 2013) to chilling. Nevertheless, for temperate and boreal trees, budburst timing predictions are not often improved by incorporating chilling requirements (Hunter and Lechowicz, 1992; Hannerz, 1999; Linkosalo, 2000; Linkosalo et al., 2008; Basler, 2016). This is due to lack of understanding of species-specific dormancy release and budburst processes and underlying mechanisms (Hannerz, 1999; Chuine, 2000; Harrington et al., 2010; Guak and Neilsen, 2013).

Insufficient chilling as a result of rising winter temperatures associated with climate warming has become a concern among researchers (Cannell and Smith, 1986; Arora et al., 2003; Yu et al., 2010; Polgar and Primack, 2011; Laube et al., 2014; Guo et al., 2015). Although earlier budburst associated with warming spring temperatures has been observed in many species in Europe (Menzel, 2000; Chmielewski and Rötzer, 2001; Chmielewski et al., 2004; Schaber and Badeck, 2005) and North America (Beaubien and Hamann, 2011; Ault et al., 2013; Ellwood et al., 2013; Primack and Gallinat, 2016), cases of delayed budburst have also been reported (Zhang et al., 2007; Ibáñez et al., 2010; Yu et al., 2010; Cook et al., 2012). The phenological responses to further warming are predicted to accelerate (Morin et al., 2009) or slow down (Fu et al., 2015). These mixed responses to warming likely reflect genetic and geographic variations in species chilling satisfaction under the current and future climate (Howe et al., 2003; Zhang et al., 2007; Luedeling et al., 2013). A better understanding of the effects of chilling deficiency on trees is, therefore, needed to reduce uncertainty on how climate warming and reduced chilling accumulation may affect tree phenology and growth, forest productivity, and forest ecosystems.

White birch (*Betula papyrifera* Marsh.), balsam poplar (*Populus balsamifera* L.), trembling aspen (*Populus tremuloides* Michx.), black spruce [*Picea mariana* (Mill.) B.S.P.], white spruce [*Picea glauca* (Moench) Voss], jack pine (*Pinus banksiana* Lamb.), and lodgepole pine (*Pinus contorta* Dougl. ex. Loud.) are widely distributed in boreal forests of North America (Rowe, 1972; Burns and Honkala, 1990). As well as being critical components of boreal ecosystems (Soja et al., 2007), they are also the main tree species used for various timber products. Although spring warming is generally considered critical for budburst in northern climates (Heide, 1993; Myking and Heide, 1995; Colombo, 1998; Linkosalo et al., 2008), chilling requirements or chilling–heat relationships have not been quantified for these tree species. In this study, we investigated the effects of insufficient chilling on budburst and shoot growth of seven northern forest tree species under different exposures to natural chilling. We hypothesized that (1) chilling requirements and chilling–heat relationships for dormancy release and budburst would be species-specific (Polgar and Primack, 2011; Polgar et al., 2014), and, therefore, (2) the effects of **i**nsufficient chilling would differ among species and so would their responses to possible reduced chilling accumulation under a warming climate.

## Materials and Methods

### Seedlings

Seeds of broadleaf species that were collected from open-pollinated trees at the Petawawa Research Forest (46°00N, 77°42′W) (white birch and trembling aspen) and at Kemptville (45°02′N, 75°39W) (balsam poplar), ON, Canada, were provided by the National Tree Seed Centre at Fredericton, NB, Canada. Seeds were sown into 3.8 × 21 cm SC-10 Super Cell tubes filled with 2:1 peat moss/vermiculite (*v*/*v*) mixture in late June 2010 and grown in a greenhouse at the Ontario Forest Research Institute in Sault Ste. Marie, ON, Canada (46° 30′N, 84° 20′W). The greenhouse was programmed to provide 26°C (day)/18°C (night) temperatures and a 16-h photoperiod. Seedlings were watered as required and fertilized weekly with 20-8-20 (N-P-K) (Plant Products Co., Ltd, Brampton, ON, Canada) at 100 ppm N. Beginning early September 2010, seedlings were moved outdoors and fertilization was adjusted to 20-20-20 at 50 ppm N. Starting in mid-October, fertilization was discontinued but seedlings were watered as needed. By the end of November, leaves had abscised from all seedlings. In mid-December, seedlings were sealed in plastic bags, boxed, and stored in a freezer at -3°C. In mid-March, after 3 months in frozen storage, seedlings were moved to refrigerated storage at 2°C. In early May, seedlings were removed from the boxes and transplanted into 15-cm diameter pots. The transplanted seedlings were grown outside under natural environmental conditions for 1 year, and watered and fertilized as needed during the growing season.

Lodgepole pine seedlings were initially grown in containers in a greenhouse at Tree Time Services Inc./Coast to Coast Reforestation in Smoky Lake facility, Smoky Lake, AB, Canada, from open-pollinated seeds collected from the area southwest of Whitecourt, AB, Canada (54° 04′N, 116° 41′W). Black spruce, white spruce, and jack pine seedlings were initially grown in containers at the Millson Forestry Service Inc. at Timmins, ON, Canada, with seeds obtained from tree improvement seed orchards established for the nearby Martel Forest (47°50′–48°28′N, and 82°15′–83°25′W). Following overwinter storage, seedlings were shipped to Sault Ste Marie, ON, Canada. Upon arrival in early June 2013, the 1-year-old container seedlings were transplanted into 4″ square (10 cm side × 15 cm deep) pots filled with 2:1 peat moss/vermiculite (*v/v*) mixture and grown in a greenhouse at the Ontario Forest Research Institute. The greenhouse received natural photoperiods with temperatures 2 to 5°C above ambient conditions. Seedlings were watered as required and fertilized weekly with 20-8-20 (N-P-K) (Plant Products Co., Ltd, Brampton, ON, Canada) at 100 ppm N for a month before being moved outdoors in early July. Watering and fertilization continued as required until early September when fertilization was adjusted to 20-20-20 at 50 ppm N and then discontinued in mid-October.

Both broadleaf and conifer seedlings remained outdoors to undergo natural hardening and dehardening under ambient photoperiod and temperatures. Average seedling height at the outset of budburst experiments in the fall of 2014 was 121 cm for trembling aspen, 77 cm for balsam poplar, 142 cm for white birch, 54 cm for black spruce, 43 cm for white spruce, 44 cm for jack pine, and 36 cm for lodgepole pine.

### Budburst Experiments

**FIGURE 1:**
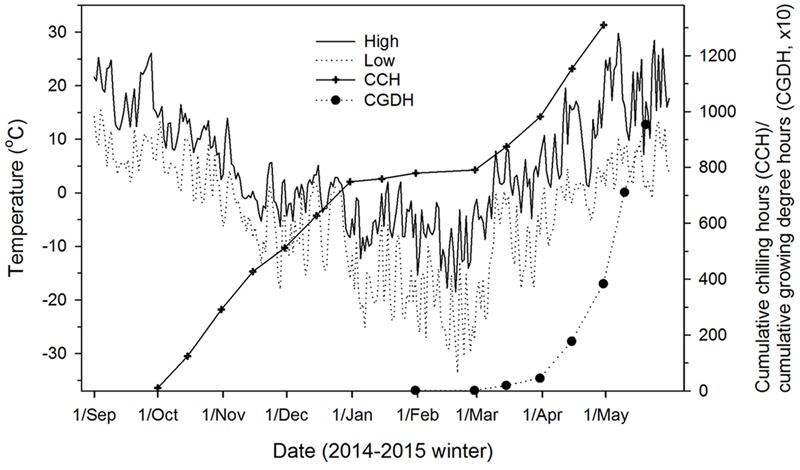
Outdoor air temperatures (daily high and low), chilling accumulation (cumulative weighted chilling hours as computed by Hänninen, 1990), and heat accumulation (cumulative growing degree hours > 0°C since January 1) at the study site (Sault Ste. Marie, ON, Canada) where seedlings were exposed to ambient temperatures from fall 2014 to spring 2015.

A series of budburst experiments were carried out to produce different levels of chilling fulfillment and quantify the corresponding heat requirement for budburst and the subsequent quality of budburst and shoot growth. The experiments were conducted in two computer-controlled walk-in climate chambers (i.e., two replications) set at 15°C (day)/5°C (night) and a 14-h photoperiod at 350 μmol m^-2^ s^-1^ photosynthetic photon flux density (PPFD). This temperature and photoperiod regime is typical of late April and early May conditions in northern Ontario, which is when leaf out generally occurs in the study area (**Figure 1**). Batches of seedlings were sequentially moved into the climate chambers from outdoors at 10-day intervals between October 1st and January 1st and 20-day intervals thereafter to mid-April, which resulted in a time series of 16 budburst experiments for each species (15 in the climate chambers and 1 outdoors). Seedlings going into these 16 budburst experiments received different levels of chilling as they were continuously exposed to outdoor chilling temperatures before being forced to burst buds in the climate chambers. When outdoor temperatures were below -5°C, to minimize temperature shock seedlings were covered in large plastic bags and kept at 0°C overnight before their transfer to climate chambers. In each of the budburst experiments, eight seedlings from each conifer species and four seedlings from each broadleaf species were placed in each of the two climate chambers (replicates). The total number of seedlings used was 256 for each conifer species and 128 for each broadleaf species, except for balsam poplar which had 110 seedlings.

### Data Collection

Budburst was assessed daily during the experiments. A bud was considered to have flushed when its scales were broken and new green foliage was clearly visible. Budburst was considered abnormal if it started first in lower branches, stems, and roots in broadleaves and if bud and needle expansion were constrained in conifers. The time and heat accumulations required for budburst were determined for individual seedlings. For seedlings that did not flush throughout the experiments, a maximum heat accumulation was calculated from the start of the experiment to May 31 when trees growing outdoors had begun flushing. Shoot growth was assessed on individual seedlings by measuring the length of the longest shoot 30 days after budburst.

### Determining Chilling–Heat Relationships

The chilling–heat relationship was determined by species with hourly temperature data from a HOBO weather station (three sensors set at 0.80 m height) installed beside the outdoor seedlings and records from inside climate chambers. The chilling accumulation was calculated using the Sarvas Chilling Rate Model (Sarvas, 1974) which was reformulated by Hänninen (1990) and Kramer (1994),

$$CH=\{\begin{array}{c} 0.159T+0.506,\;\;-3.4<T\leq +3.5\\ -0.159T+1.621,\;\;\;3.5<T\leq +10.4\\ \;0\;\;\;\;\;\;\;\;\;\;\;\;\;\;\mathrm{otherwise} \end{array}$$

where *CH* is weighted chilling hours, *T* is the actual temperature imposed on seedlings, and -3.4, 3.5, and 10.4°C are three threshold values for lower, optimum, and upper limits of chilling rate. Because of the possible negative effect of high temperatures on the effectiveness of chilling (Young, 1992; Arora et al., 2003), chilling accumulation was calculated from late September to the time of transfer to the climate chambers. Similarly, possible chilling in the climate chambers under cooler night temperature (5°C for 8 h) was not considered. For the budburst experiment with outdoor seedlings, chilling accumulation continued until late April when outdoor daily temperature was generally below 15°C. As jack pine, lodgepole pine, and balsam poplar started budburst before late April, their chilling accumulation to the time of budburst was calculated for individual trees.

The accumulation of heat (expressed as cumulative growing degree hours above 0°C; see Man and Lu, 2010) included high temperatures in the climate chambers and under outdoor conditions between March 1 and the time seedlings were brought into the climate chambers (**Figure 1**).

**FIGURE 2:**
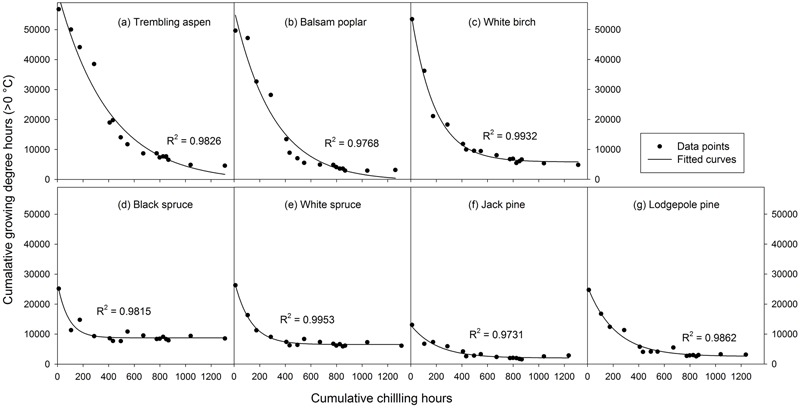
Exponential curves fitted with 16 mean heat requirements (cumulative growing degree hours > 0°C) and corresponding chilling accumulations (cumulative weighted chilling hours since September 29) of budburst experiments conducted sequentially from October 1 (left, climate chambers) to May (right, outdoor conditions) for seven tree species (a–g).

The relationship between chilling accumulation and heat requirement for budburst was investigated by fitting a 3-parameter exponential decay curve following Cannell and Smith (1983) and Hannerz et al. (2003) in the form:

$$y=a+be^{-cx}$$

where *y* is the heat requirement for budburst, *x* is the chilling accumulation (i.e., cumulative weighted chilling hours), *e* is the base of natural logarithm, and *a, b*, and *c* are the model parameters. The parameter *c* reflects the rate of chilling completion, i.e., a greater value indicates earlier and faster completion of chilling (**Figure 2**). We used the method documented by Hannerz et al. (2003) to determine chilling requirements for budburst, which was set as 1.05^∗^*a*, a point slightly before the lowest value of the fitted curves (**Figure 2**). This method did not work well for trembling aspen and balsam poplar as they had negative estimates for parameter *a*. For these species, *a* was estimated as the lowest value from the observed heat requirement for budburst.

### Data Analysis

Heat requirement for budburst and shoot growth of individual seedlings were subjected to a 2-way analysis of variance based on the linear model:

$$y_{ijkl}=\mu +E_{i}+S_{j}+ES_{ij}+\varepsilon_{ijkl}$$

where y_ijkl_ is the observation of the l^th^ seedling of the j^th^ species tested in the k^th^ replication of the i^th^ experiment; μ is the overall mean; E_i_ is the i^th^ experiment; S_j_ is the j^th^ species; ES_ij_ is the interaction between i^th^ experiment and j^th^ species; and 𝜀_ijkl_ is the residual. One-way ANOVA was performed to test differences among tree species in the rate of chilling completion (parameter *c* of the exponential curve) and chilling requirement determined from chilling–heat relationships for tree species and replications (individual climate chambers). Multiple contrasts were conducted to examine differences in heat requirement for budburst and shoot growth among experiments within a species or among species within an experiment, and in rate of chilling completion and chilling requirement among species. The *P*-values were corrected using Tukey’s method in SAS 9.3 (SAS Institute Inc., 2011).

## Results

### Chilling and Heat Requirements

Analysis of variance indicated that both budburst experiments (i.e., timing) and tree species significantly affected heat requirement for budburst (*p* < 0.001 for species, budburst experiments, or species by experiment interaction). The amount of heat required for budburst decreased progressively with experiment start time in all species, and was greater in broadleaf than conifer species in experiments conducted before December 10, and greater in spruce than pine across almost all budburst experiments (see Supplementary Table S1). Among species, the change in heat requirement became insignificant over time, occurring first for black spruce and white spruce (by October 31) and last for trembling aspen (by December 20) (**Figure 2** and Supplementary Table S1).

The rate of chilling completion (i.e., estimate of parameter *c* in the fitted exponential curves) was highest for black spruce and white spruce and lowest for trembling aspen and balsam poplar, with intermediate values for white birch, jack pine, and lodgepole pine (**Figure 2** and Supplementary Table S2). The chilling requirement (amount of chilling accumulation when chilling need was fully met) was less than 600 chilling hours (prior to January 1) for black spruce and white spruce and over 1100 chilling hours for trembling aspen and lodgepole pine. Balsam poplar, white birch, and jack pine were intermediate, completing their chilling by late March (about 900 chilling hours).

The heat requirement for budburst after the completion of chilling, determined from the fitted exponential curves and expressed as cumulative growing degree hours was, in descending order, 9204 for black spruce, 6831 for white spruce, 6109 for white birch, 4816 for trembling aspen, 3062 for balsam poplar, 2691 for lodgepole pine, and 2114 for jack pine (**Figure 2** and Supplementary Table S2).

### Effects of Insufficient Chilling

**FIGURE 3:**
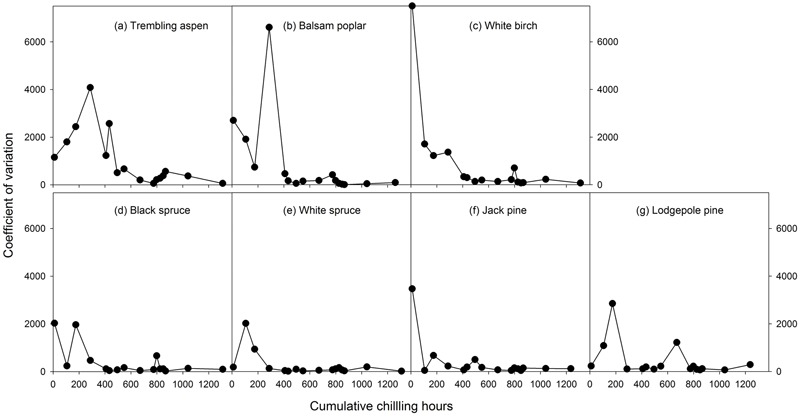
The relationships between chilling accumulation (cumulative weighted chilling hours since September 29) and coefficient variations of heat requirements among individual trees from 16 budburst experiments conducted sequentially from October 1 (left, climate chambers) to May (right, outdoor conditions) for seven tree species (a–g).

**FIGURE 4:**
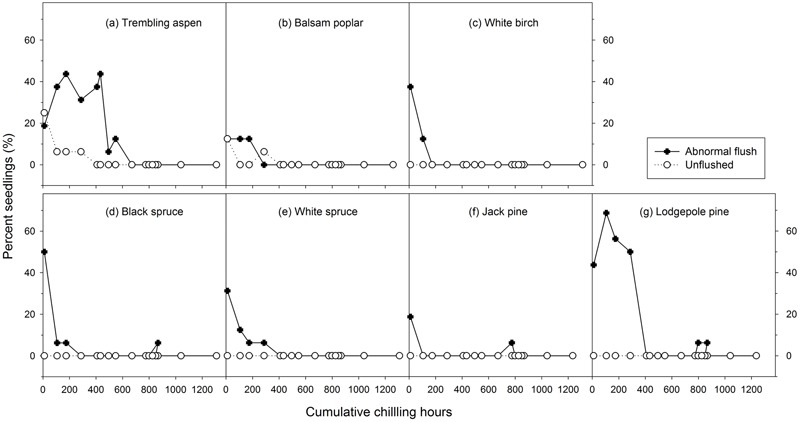
The relationships between chilling accumulation (cumulative weighted chilling hours since September 29) and budburst quality (percent abnormally flushed and unflushed seedlings) from 16 budburst experiments conducted sequentially from October 1 (left, climate chambers) to May (right, outdoor conditions) for seven tree species (a–g).

**FIGURE 5:**
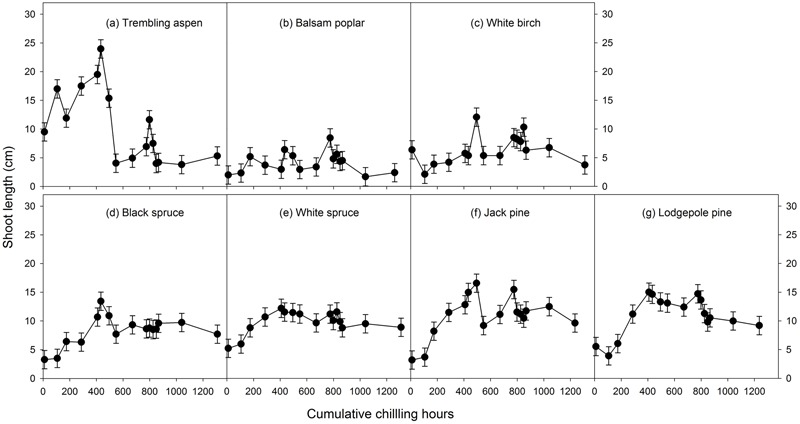
The relationships between chilling accumulation (cumulative weighted chilling hours since September 29) and shoot growth (length of longest new shoot within a month of budburst, least square means ± SE) from 16 budburst experiments conducted sequentially from October 1 (left, climate chambers) to May (right, outdoor conditions) for seven tree species (a–g).

Mean heat requirements and coefficient of variations (CV) were substantially higher before chilling requirements were fulfilled, especially in broadleaves (**Figures 2, 3** and Supplementary Tables S1, S2). Budburst experiments started before the end of November, when chilling accumulation was < 500 chilling hours, produced abnormal budburst in broadleaves, which typically included early and erratic burst of buds in lower branches, as well as the burst of basal buds at lower stems and on roots (aspen only); some aspen and balsam poplar had not burst buds after 243 days in the climate chambers (**Figure 4**). Among the three broadleaf species, trembling aspen had significantly longer new shoots (from lower stem sprouting and suckering) in the fall budburst experiments (<500 chilling hours) than the winter and spring budburst experiments (>500 chilling hours) compared to balsam poplar and white birch (*p* < 0.01 for species, budburst experiments, or species by experiment interaction, see **Figure 5**).

Insufficient chilling also delayed the time of budburst, increased variations among individual trees, and caused abnormal budburst in conifers (**Figures 2–4** and Supplementary Table S1). The typical patterns of abnormal budburst in conifers included constrained bud expansion, shortened new needle length, and shorter terminal than lateral shoots. The effect was strongest in lodgepole pine and weakest in jack pine, with the spruces intermediate. Insufficient chilling did not, however, stop conifers from bursting bud, but when chilling accumulation was less than 300 chilling hours significantly shortened the length of new shoots (**Figure 5**).

## Discussion

Our hypotheses were generally true in terms of species chilling and heat needs, and responses to insufficient chilling. Among the seven species examined, chilling of black spruce and white spruce was completed early in the year (before January 1), which was consistent with findings reported by Hannerz et al. (2003) for Norway spruce (*Picea abies*) in Europe and the assumption by Colombo (1998) for white spruce in Ontario. Cannell and Smith (1983) reviewed chilling requirements of several species and noticed that both white spruce and Norway spruce chilling requirements were completed earlier and faster than those of other tree species, including Sitka spruce (*Picea sitchensis*), Douglas fir (*Pseudotsuga menziesii*), and eastern cottonwood (*Populus deltoides*).

The estimated chilling requirements for broadleaf and pine trees are also generally comparable to results for similar species reported in the literature. For example, Laube et al. (2014) showed that *Betula pendula* and *Populus tremula*—tree species in the same genera as white birch and trembling aspen—needed more chilling than four pine species (*Pinus nigra, Pinus strobus, Pinus sylvestris*, and *Pinus wallichiana*). In this study, lodgepole pine had a much higher rate of abnormal budburst than the other conifers when chilling accumulation was < 400 chilling hours, which was consistent with the observation by Sloan (1991) in ID, United States, that chilling requirements for lodgepole pine were not fulfilled by the end of March.

Insufficient chilling delayed budburst, as suggested by others (Cannell and Smith, 1983, 1986; Myking and Heide, 1995; Heide, 2003; Morin et al., 2009; Laube et al., 2014; Fu et al., 2015), but only extremely low chilling hours affected normal budburst, as was also shown by Arora et al. (2003) and Harrington et al. (2010). Chilling accumulation levels that resulted in abnormal budburst and growth in boreal trees seem to be related to their chilling requirements, which are slightly higher in trembling aspen and lodgepole pine (<400 chilling hours) than the other five species studied (<200 to 300 chilling hours). Comparatively, insufficient chilling had more effect on budburst and growth of the broadleaf trees than the conifers. The abnormal burst of lower stem basal buds and the initiation of root suckers, due to their shallower dormancy (Rinne et al., 1994; Heide, 2011), may result in multiple stems and reduce the value of broadleaf trees for timber products.

In this study, low chilling needs were often associated with high heat requirements for budburst (i.e., black spruce and white spruce) and vice versa (i.e., trembling aspen and lodgepole pine), as was also found by Luedeling et al. (2013) for chestnut (*Castanea* spp.), cherry (*Prunus* spp.), and walnut (*Juglans* spp.). This may reflect different strategies in boreal species to prevent precocious spring development. High chilling and low heat requirements are advantageous for avoiding early dormancy release, but prompt new growth as long as conditions are suitable, whereas high heat requirements help prevent early start of new growth when spring frosts are common. Comparatively, the combinations of low chilling with low heat and high chilling with high heat would be disadvantageous, as they could lead to extremely early budburst and high risk of cold damage or considerable delays in budburst and reduced opportunity for growth when weather conditions are favorable.

The northern Ontario climate is characterized by long and cold winters. Contributions to chilling were nearly zero during January/February in winter 2014–2015. Climatic warming would enhance chilling contribution from these freezing winter months (Harrington and Gould, 2015) and chilling accumulation could remain at the current level if temperatures increase uniformly (Heide, 1993; Myking and Heide, 1995) or increase if greater warming occurs in winter than in fall and spring. In either case, time to budburst would be earlier due to greater heat accumulation in spring, as has been observed elsewhere (Menzel, 2000; Schaber and Badeck, 2005; Zhang et al., 2007; Beaubien and Hamann, 2011), especially for black spruce and white spruce which have low chilling requirements. However, trees may not be able to fully use the extended growing season as risk of freezing damage may increase with increasing temperature variability (Gu et al., 2008; Rigby and Porporato, 2008; Man et al., 2009, 2013; Beaubien and Hamann, 2011; Augspurger, 2013). If warming reduces chilling accumulation (i.e., greater warming in fall and spring than in winter), species with high chilling requirements, such as trembling aspen and lodgepole pine, may be affected (Cannell and Smith, 1986; Morin et al., 2009; Hänninen and Tanino, 2011; Polgar and Primack, 2011; Laube et al., 2014). Results from this study indicate that the effects depend not only on species’ chilling requirements, but also on their chilling–heat relationships. Therefore, mixed phenological responses to warming are more likely to occur (Zhang et al., 2007). A 30% reduction in chilling accumulation from the current level (1310 chilling hours in the study area for winter 2014–2015) would delay budburst of trembling aspen for 4 days and that of lodgepole pine, jack pine, and white birch for less than 1 day (for an early summer day of 20°C day/15°C night in Sault Ste. Marie, ON, Canada; see Environment Canada online archive at http://climate.weather.gc.ca). A further reduction to 50% would delay budburst for 20 days in trembling aspen, 9 days in balsam poplar, 3 days in white birch and lodgepole pine, and 1 day in jack pine. A 70 to 80% reduction in chilling accumulation would be required before normal budburst and growth of new needle and shoots are affected. The actual effects on budburst and growth, however, would be less due to compensation of insufficient chilling by greater spring heat accumulation under predicted future climate (Menzel, 2000; Schaber and Badeck, 2005; Beaubien and Hamann, 2011; Fu et al., 2015) and would vary from year to year due to inter-annual temperature variations that affect both chilling and heat accumulations (1310 vs. 1390 chilling hours for 2014–2015 and 2015–2016 winters, respectively, in the study area; also see Bailey and Harrington, 2006). Due to uncertainty about future climate, possible within-species variations in chilling requirements and chilling–heat relationships by genetic differentiation among populations (Howe et al., 2003; Hawkins and Dhar, 2012), and ontogenic differences between seedlings and mature trees (Vitasse, 2013), caution is required when applying these results.

## Author Contributions

Conceived and designed the experiments: RM, PL, and Q-LD. Performed the experiments: RM and PL. Analyzed the data: RM and PL. Wrote the paper: RM, PL, and Q-LD.

## Conflict of Interest Statement

The authors declare that the research was conducted in the absence of any commercial or financial relationships that could be construed as a potential conflict of interest.
