# Supplementary material for: Insufficient Chilling Effects Vary among Boreal Tree Species and Chilling Duration
Source: Front Plant Sci. 2017 Aug 15;8:1354. doi: 10.3389/fpls.2017.01354 (PMC5559465; doi:10.3389/fpls.2017.01354)
Supplement: Supplementary file 1 [file Table_1.docx]

**TABLE S1:** Results of multiple comparisons of heat requirements for budburst: a) differences among budburst experiments within a species, where + and = indicate experimental mean is significantly greater than or statistically insignificant from, respectively, the combined mean of subsequent experiments; and b) differences between species groups within an experiment, B:C – broadleaves versus conifers and S:P – spruce versus pine species, where + and = indicate B (or S) is statistically greater than or equal to C (or P). Date and chilling indicate the time that each budburst experiment started and associated chilling accumulation. The symbol Outd# refers to chilling accumulations of outdoor (Outd) stored seedlings, which ranged from 1234 to 1308 chilling hours due to difference in time of budburst among species.

| Experiments | 1 | 2 | 3 | 4 | 5 | 6 | 7 | 8 | 9 | 10 | 11 | 12 | 13 | 14 | 15 | 16 |
| --- | --- | --- | --- | --- | --- | --- | --- | --- | --- | --- | --- | --- | --- | --- | --- | --- |
| Date | Oct1 | Oct11 | Oct21 | Oct31 | Nov10 | Nov20 | Nov30 | Dec10 | Dec20 | Dec30 | Jan9 | Jan29 | Feb18 | Mar10 | Mar30 | Outd |
| Chilling | 10 | 106 | 174 | 286 | 408 | 433 | 494 | 547 | 670 | 775 | 799 | 825 | 849 | 867 | 1040 | Outd# |
| Sequential changes of heat requirements among budburst experiments | | | | | | | | | | | | | | | | |
| AT | + | + | + | + | + | + | + | + | **=** | **=** | **=** | **=** | **=** | **=** | **=** | **=** |
| PB | + | + | + | + | + | **=** | **=** | **=** | **=** | **=** | **=** | **=** | **=** | **=** | **=** | **=** |
| BW | + | + | + | + | + | **=** | **=** | **=** | **=** | **=** | **=** | **=** | **=** | **=** | **=** | **=** |
| SB | + | **=** | + | **=** | **=** | **=** | **=** | **=** | **=** | **=** | **=** | **=** | **=** | **=** | **=** | **=** |
| SW | + | + | + | **=** | **=** | **=** | **=** | **=** | **=** | **=** | **=** | **=** | **=** | **=** | **=** | **=** |
| PJ | + | + | + | + | **=** | **=** | **=** | **=** | **=** | **=** | **=** | **=** | **=** | **=** | **=** | **=** |
| PL | + | + | + | + | **=** | **=** | **=** | **=** | **=** | **=** | **=** | **=** | **=** | **=** | **=** | **=** |
| Differences between species groups | | | | | | | | | | | | | | | | |
| B:C | + | + | + | + | + | + | + | **=** | **=** | **=** | **=** | **=** | **=** | **=** | **=** | **=** |
| S:P | + | **=** | + | **=** | + | + | + | + | + | + | + | + | + | + | + | + |

Note: Species abbreviations are: AT – trembling aspen, PB – balsam poplar, BW – white birch, SB – black spruce, SW – white spruce, PJ – jack pine, and PL – lodgepole pine.
