# Supplementary material for: Insufficient Chilling Effects Vary among Boreal Tree Species and Chilling Duration
Source: Front Plant Sci. 2017 Aug 15;8:1354. doi: 10.3389/fpls.2017.01354 (PMC5559465; doi:10.3389/fpls.2017.01354)
Supplement: Supplementary file 2 [file Table_2.DOCX]

**TABLE S2:** Parameter estimates for an exponential decay curve (*a, b*, and *c*) illustrating the relationship between chilling accumulation (cumulative weighted chilling hours) and heat requirement (cumulative growing degree hours >0 °C) for budburst in the 7 tree species examined.

| Species | a | b | c | Chilling requirement (chilling hours) |
| --- | --- | --- | --- | --- |
| Trembling aspen | -703 | 63495 | 0.0025c | 1246a |
| Balsam poplar | -546 | 57231 | 0.0032c | 991ab |
| White birch | 5819 | 50394 | 0.0054b | 955ab |
| Black spruce | 8768 | 18145 | 0.0118a | 316b |
| White spruce | 6505 | 21558 | 0.0080ab | 524ab |
| Jack pine | 2013 | 10993 | 0.0049b | 958ab |
| Lodgepole pine | 2563 | 23263 | 0.0046b | 1131a |

ANOVA statistical difference <0.01 0.035

Note: The chilling requirements of trembling aspen and balsam poplar were obtained from the lowest observed heat requirement for budburst. The corresponding heat requirements (cumulative growing degree hours >0 °C) at completion of chilling requirements for the species listed in the table are, from top to bottom, 4816, 3062, 6109, 9204, 6831, 2114, and 2691.
